# Assessing the impact of healthcare research: A systematic review of methodological frameworks

**DOI:** 10.1371/journal.pmed.1002370

**Published:** 2017-08-09

**Authors:** Samantha Cruz Rivera, Derek G. Kyte, Olalekan Lee Aiyegbusi, Thomas J. Keeley, Melanie J. Calvert

**Affiliations:** Centre for Patient Reported Outcomes Research, Institute of Applied Health Research, College of Medical and Dental Sciences, University of Birmingham, Birmingham, United Kingdom; Queens University Belfast, UNITED KINGDOM

## Abstract

**Background:**

Increasingly, researchers need to demonstrate the impact of their research to their sponsors, funders, and fellow academics. However, the most appropriate way of measuring the impact of healthcare research is subject to debate. We aimed to identify the existing methodological frameworks used to measure healthcare research impact and to summarise the common themes and metrics in an impact matrix.

**Methods and findings:**

Two independent investigators systematically searched the Medical Literature Analysis and Retrieval System Online (MEDLINE), the Excerpta Medica Database (EMBASE), the Cumulative Index to Nursing and Allied Health Literature (CINAHL+), the Health Management Information Consortium, and the Journal of Research Evaluation from inception until May 2017 for publications that presented a methodological framework for research impact. We then summarised the common concepts and themes across methodological frameworks and identified the metrics used to evaluate differing forms of impact. Twenty-four unique methodological frameworks were identified, addressing 5 broad categories of impact: (1) ‘primary research-related impact’, (2) ‘influence on policy making’, (3) ‘health and health systems impact’, (4) ‘health-related and societal impact’, and (5) ‘broader economic impact’. These categories were subdivided into 16 common impact subgroups. Authors of the included publications proposed 80 different metrics aimed at measuring impact in these areas. The main limitation of the study was the potential exclusion of relevant articles, as a consequence of the poor indexing of the databases searched.

**Conclusions:**

The measurement of research impact is an essential exercise to help direct the allocation of limited research resources, to maximise research benefit, and to help minimise research waste. This review provides a collective summary of existing methodological frameworks for research impact, which funders may use to inform the measurement of research impact and researchers may use to inform study design decisions aimed at maximising the short-, medium-, and long-term impact of their research.

## Abstract 2

Derek Kyte and colleagues systematically review approaches to the evaluation of health research.

## Author summary

**Why was this study done?:**

- There is a growing interest in demonstrating the impact of research in order to minimise research waste, allocate resources efficiently, and maximise the benefit of research. However, there is no consensus on which is the most appropriate tool to measure the impact of research.
- To our knowledge, this review is the first to synthesise existing methodological frameworks for healthcare research impact, and the associated impact metrics by which various authors have proposed impact should be measured, into a unified matrix.

**What did the researchers do and find?:**

- We conducted a systematic review identifying 24 existing methodological research impact frameworks.
- We scrutinised the sample, identifying and summarising 5 proposed impact categories, 16 impact subcategories, and over 80 metrics into an impact matrix and methodological framework.

**What do these findings mean?:**

- This simplified consolidated methodological framework will help researchers to understand how a research study may give rise to differing forms of impact, as well as in what ways and at which time points these potential impacts might be measured.
- Incorporating these insights into the design of a study could enhance impact, optimizing the use of research resources.

## Introduction

In 2010, approximately US$240 billion was invested in healthcare research worldwide [1]. Such research is utilised by policy makers, healthcare providers, and clinicians to make important evidence-based decisions aimed at maximising patient benefit, whilst ensuring that limited healthcare resources are used as efficiently as possible to facilitate effective and sustainable service delivery. It is therefore essential that this research is of high quality and that it is impactful—i.e., it delivers demonstrable benefits to society and the wider economy whilst minimising research waste [1,2]. Research impact can be defined as ‘any identifiable ‘benefit to, or positive influence on the economy, society, public policy or services, health, the environment, quality of life or academia’ (p. 26) [3].

There are many purported benefits associated with the measurement of research impact, including the ability to (1) assess the quality of the research and its subsequent benefits to society; (2) inform and influence optimal policy and funding allocation; (3) demonstrate accountability, the value of research in terms of efficiency and effectiveness to the government, stakeholders, and society; and (4) maximise impact through better understanding the concept and pathways to impact [4–7].

Measuring and monitoring the impact of healthcare research has become increasingly common in the United Kingdom [5], Australia [5], and Canada [8], as governments, organisations, and higher education institutions seek a framework to allocate funds to projects that are more likely to bring the most benefit to society and the economy [5]. For example, in the UK, the 2014 Research Excellence Framework (REF) has recently been used to assess the quality and impact of research in higher education institutions, through the assessment of impact cases studies and selected qualitative impact metrics [9]. This is the first initiative to allocate research funding based on the economic, societal, and cultural impact of research, although it should be noted that research impact only drives a proportion of this allocation (approximately 20%) [9].

In the UK REF, the measurement of research impact is seen as increasingly important. However, the impact element of the REF has been criticised in some quarters [10,11]. Critics deride the fact that REF impact is determined in a relatively simplistic way, utilising researcher-generated case studies, which commonly attempt to link a particular research outcome to an associated policy or health improvement despite the fact that the wider literature highlights great diversity in the way research impact may be demonstrated [12,13]. This led to the current debate about the optimal method of measuring impact in the future REF [10,14]. The Stern review suggested that research impact should not only focus on socioeconomic impact but should also include impact on government policy, public engagement, academic impacts outside the field, and teaching to showcase interdisciplinary collaborative impact [10,11]. The Higher Education Funding Council for England (HEFCE) has recently set out the proposals for the REF 2021 exercise, confirming that the measurement of such impact will continue to form an important part of the process [15].

With increasing pressure for healthcare research to lead to demonstrable health, economic, and societal impact, there is a need for researchers to understand existing methodological impact frameworks and the means by which impact may be quantified (i.e., impact metrics; see Box 1, 'Definitions’) to better inform research activities and funding decisions. From a researcher’s perspective, understanding the optimal pathways to impact can help inform study design aimed at maximising the impact of the project. At the same time, funders need to understand which aspects of impact they should focus on when allocating awards so they can make the most of their investment and bring the greatest benefit to patients and society [2,4,5,16,17].

**Box 1. Definitions**

- **Research impact:** ‘any identifiable benefit to, or positive influence on, the economy, society, public policy or services, health, the environment, quality of life, or academia’ (p. 26) [3].
- **Methodological framework:** ‘a body of methods, rules and postulates employed by a particular procedure or set of procedures (i.e., framework characteristics and development)’ [18].
- **Pathway:** ‘a way of achieving a specified result; a course of action’ [19].
- **Quantitative metrics:** ‘a system or standard of [quantitative] measurement’ [20].
- **Narrative metrics:** ‘a spoken or written account of connected events; a story’ [21].

Whilst previous researchers have summarised existing methodological frameworks and impact case studies [4,22–27], they have not summarised the metrics for use by researchers, funders, and policy makers. The aim of this review was therefore to (1) identify the methodological frameworks used to measure healthcare research impact using systematic methods, (2) summarise common impact themes and metrics in an impact matrix, and (3) provide a simplified consolidated resource for use by funders, researchers, and policy makers.

## Methods

### Search strategy and selection criteria

Initially, a search strategy was developed to identify the available literature regarding the different methods to measure research impact. The following keywords: ‘Impact’, ‘Framework’, and ‘Research’, and their synonyms, were used during the search of the Medical Literature Analysis and Retrieval System Online (MEDLINE; Ovid) database, the Excerpta Medica Database (EMBASE), the Health Management Information Consortium (HMIC) database, and the Cumulative Index to Nursing and Allied Health Literature (CINAHL+) database (inception to May 2017; see S1 Appendix for the full search strategy). Additionally, the nonindexed Journal of Research Evaluation was hand searched during the same timeframe using the keyword ‘Impact’. Other relevant articles were identified through 3 Internet search engines (Google, Google Scholar, and Google Images) using the keywords ‘Impact’, ‘Framework’, and ‘Research’, with the first 50 results screened. Google Images was searched because different methodological frameworks are summarised in a single image and can easily be identified through this search engine. Finally, additional publications were sought through communication with experts.

Following Preferred Reporting Items for Systematic Reviews and Meta-Analyses (PRISMA) guidelines (see S1 PRISMA Checklist), 2 independent investigators systematically screened for publications describing, evaluating, or utilising a methodological research impact framework within the context of healthcare research [28]. Papers were eligible if they included full or partial methodological frameworks or pathways to research impact; both primary research and systematic reviews fitting these criteria were included. We included any methodological framework identified (original or modified versions) at the point of first occurrence. In addition, methodological frameworks were included if they were applicable to the healthcare discipline with no need of modification within their structure. We defined ‘methodological framework’ as ‘a body of methods, rules and postulates employed by a particular procedure or set of procedures (i.e., framework characteristics and development)’ [18], whereas we defined ‘pathway’ as ‘a way of achieving a specified result; a course of action’ [19]. Studies were excluded if they presented an existing (unmodified) methodological framework previously available elsewhere, did not explicitly describe a methodological framework but rather focused on a single metric (e.g., bibliometric analysis), focused on the impact or effectiveness of interventions rather than that of the research, or presented case study data only. There were no language restrictions.

### Data screening

Records were downloaded into Endnote (version X7.3.1), and duplicates were removed. Two independent investigators (SCR and OLA) conducted all screening following a pilot aimed at refining the process. The records were screened by title and abstract before full-text articles of potentially eligible publications were retrieved for evaluation. A full-text screening identified the publications included for data extraction. Discrepancies were resolved through discussion, with the involvement of a third reviewer (MJC, DGK, and TJK) when necessary.

### Data extraction and analysis

Data extraction occurred after the final selection of included articles. SCR and OLA independently extracted details of impact methodological frameworks, the country of origin, and the year of publication, as well as the source, the framework description, and the methodology used to develop the framework. Information regarding the methodology used to develop each methodological framework was also extracted from framework webpages where available. Investigators also extracted details regarding each framework’s impact categories and subgroups, along with their proposed time to impact (‘short-term’, ‘mid-term’, or ‘long-term’) and the details of any metrics that had been proposed to measure impact, which are depicted in an impact matrix. The structure of the matrix was informed by the work of M. Buxton and S. Hanney [2], P. Buykx et al. [5], S. Kuruvila et al. [29], and A. Weiss [30], with the intention of mapping metrics presented in previous methodological frameworks in a concise way. A consensus meeting with MJC, DGK, and TJK was held to solve disagreements and finalise the data extraction process.

## Results

### Included studies

**Fig 1:**
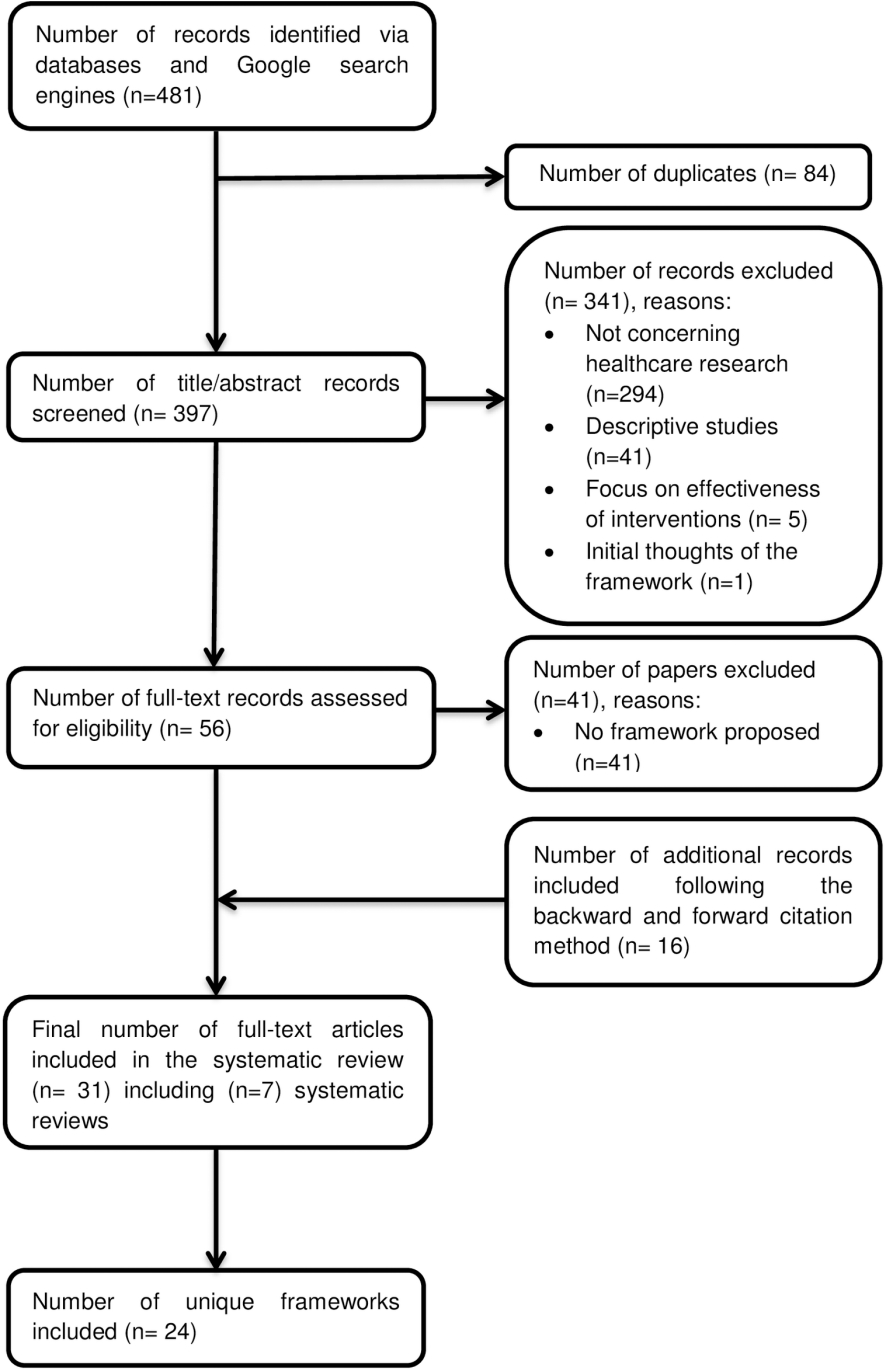
Preferred Reporting Items for Systematic Reviews and Meta-Analyses (PRISMA) flow diagram.

Our original search strategy identified 359 citations from MEDLINE (Ovid), EMBASE, CINAHL+, HMIC, and the Journal of Research Evaluation, and 101 citations were returned using other sources (Google, Google Images, Google Scholar, and expert communication) (see Fig 1) [28]. In total, we retrieved 54 full-text articles for review. At this stage, 39 articles were excluded, as they did not propose new or modified methodological frameworks. An additional 15 articles were included following the backward and forward citation method. A total of 31 relevant articles were included in the final analysis, of which 24 were articles presenting unique frameworks and the remaining 7 were systematic reviews [4,22–27]. The search strategy was rerun on 15 May 2017. A further 19 publications were screened, and 2 were taken forward to full-text screening but were ineligible for inclusion.

### Methodological framework characteristics

The characteristics of the 24 included methodological frameworks are summarised in Table 1, 'Methodological framework characteristics’. Fourteen publications proposed academic-orientated frameworks, which focused on measuring academic, societal, economic, and cultural impact using narrative and quantitative metrics [2,3,5,8,29,31–39]. Five publications focused on assessing the impact of research by focusing on the interaction process between stakeholders and researchers (‘productive interactions’), which is a requirement to achieve research impact. This approach tries to address the issue of attributing research impact to metrics [7,40–43]. Two frameworks focused on the importance of partnerships between researchers and policy makers, as a core element to accomplish research impact [44,45]. An additional 2 frameworks focused on evaluating the pathways to impact, i.e., linking processes between research and impact [30,46]. One framework assessed the ability of health technology to influence efficiency of healthcare systems [47]. Eight frameworks were developed in the UK [2,3,29,37,39,42,43,45], 6 in Canada [8,33,34,44,46,47], 4 in Australia [5,31,35,38], 3 in the Netherlands [7,40,41], and 2 in the United States [30,36], with 1 model developed with input from various countries [32].

**Table 1:** Methodological framework characteristics.

Framework		Original source for modified framework	Framework description	Impact categories	Methodology followed to develop the framework				
					Literature review	Stakeholder involvement		Methods to incorporate stakeholder views	Pilot phase
Buxton and Hanney **Payback Framework [2]** UK, 1996	• The classic/purist/knowledge-driven model • The problem-solving/engineering model • The political model • The enlightenment/percolation/limestone model • The interactive/social interaction model [48–54]		Framework used to assess health research impact through academic outputs and wider societal benefits	• Knowledge benefits • Benefits to future research and research use • Political and administrative benefits • Health sector benefits • Broader economic benefits	Yes, but no systematic approach reported	None reported		None reported	A case study approach was undertaken to determine the effectiveness of the model and exemplify the impact categories
**Canadian Institutes of Health Research (CIHR) [8]** Canada, 2005	• Payback Framework [2]		Framework designed to gauge the impact of health research and the benefits of investing in health research	• Knowledge production • Research targeting and capacity • Informing policy • Health and health sector benefits • Economic impacts	Yes, but no systematic approach reported	Meetings, presentations, and panel discussions with the Canadian government, academics, health research stakeholders, and international funding agencies		Not specified	No information reported in the main paper or on the webpage
**Canadian Academy of Health Sciences (CAHS) [33]** Canada, 2009	• Payback Framework [2] • CIHR framework [8]		Framework focused on evaluating how research activity influences decision making in order to improve health, the economy, and social benefits	• Advancing knowledge • Building capacity • Informing decision making • Health • Broad socioeconomic impact	Yes, but no systematic approach reported	Semistructured interviews were conducted to collect stakeholder, sponsor, and external expert feedback		Conceptual cluster analysis was used to analyse the data collected, which helped to capture the perspective of different stakeholders involved in the health system	Six papers were commissioned to test the applicability of the framework. After modifications, the project was sent to external review and changes were made until its approval
K. Graham et al. **Alberta Innovates—Health Solutions (AIHS) impact framework [34]** Canada, 2012	• Payback Framework [2] CAHS model [33]		A framework to measure, assess, and illustrate the relationship between research investment and impact. The ultimate goal is to contribute to the most societal benefit among the people	• Advancing knowledge • Building capacity • Informing decision making • Health • Broad socioeconomic impact • Organisational performance	No systematic approach reported	None reported		None reported	The CAHS model was assessed for applicability and feasibility through retrospective and prospective studies and other tools to map pathways to impact. The findings of the assessment provided feedback to improve the AIHS model
G. Cohen et al. [35] Australia, 2014	• Payback Framework [2] • AIHS framework [34]		To evaluate the policy and practicebenefits of research outputs, which take place later and beyond the research setting	• Scholarly outputs • Translational outputs • Policy or practice impacts • Long-term population outcomes	No systematic approach reported		Two online surveys and semistructured interviews were conducted among the primary chief investigators of the research grants included	Not specified	Case studies were summarised and presented to an experience panel, which scored the relevant impact categories of this framework
S. Kuruvilla et al. **Research Impact Framework (RIF) [29]** UK, 2006		• Payback Framework [2]	Conceptual framework to describe the possible impacts of health research outcomes	• Research-related impacts • Policy impacts • Service impacts: health and intersectorial • Societal impacts	No systematic approach reported	Semistructured interviews with principal investigators of selected projects		Thematic analysis was adopted to analyse the data. The categories of the framework were used as themes	The RIF was validated through consistency with available health research literature and empirical analysis of research projects
L. Kalucy et al. **Primary Health Care Research & Information Service (PHC RIS) [31,55]** Australia, 2007		• Payback Framework [2]	Methodology designed to assess primary healthcare research. Strong collaboration, personal relationships and the participation of practitioners, health care managers, and policy makers in the definition of the research questions and in the research process were identified as the strongest pathways to impact	• Knowledge production • Research targeting, capacity, building, and absorption • Informing policy and product development • Health and health sector benefits • Broader economic benefits • Research transfer	No systematic approach reported	Part 1: Telephone interviews were conducted among the 4 chief investigators of the included projects funded by the National Health and Medical Research Council		Data provided by the interviewees were analysed following the Payback Framework and thematic analysis approach	Parts 2–17: chief investigators completed an online questionnaire to refine the methodology to measure research impact
J. Guinea et al. **Impact Oriented Monitoring (IOM) [32]** Various countries, 2015		• Payback Framework [2]	Methodology used to identify and assess the impacts of health projects through a set of predefined categories	• Advancing knowledge • Capacity building and research targeting • Informing decision making, practice, and policy • Population health and health sector benefits • Dissemination and knowledge transfer	No systematic approach reported	A project coordinators’ survey was conducted to determine and collect all the possible public health research benefits. An end users’ survey was conducted to determine the usefulness and practicality of the results of the coordinators’ survey to measure research impact. Additionally, a scoring matrix was developed to assess project impacts		Not specified	A small sample of research projects was used to test some of the methods incorporated to measure research impact
J. Lavis et al. **Exchange model [44]** Canada, 2003		**–**	Assessment tool to measure decision-making impact of health research. Impact measures are categorised according to the level of impact to be measured and the mechanism of research uptake: producer-push, user-pull, and exchange measures	• Producer-push process • User-pull process • Exchange process	No systematic approach reported	None reported		None reported	Two examples were used to demonstrate how the assessment tool can be used
L. Meagher et al. [43] UK, 2008		• Linkage and exchange model [56]	Methodology for assessing research impact of policy and practice	• Primary knowledge producers • Knowledge users, beneficiaries, brokers, and intermediaries • Impacts or research (outcomes) • Research impact processes • Lessons learned and recommendations • Methods for identifying and assessing nonacademic research impact	Yes, but no systematic approach reported	Award holders of the UK’s Economic and Social Research Council (ESRC) within the psychology field, heads of departments, ESRC-funded principal researchers, and research users were recruited to conduct a questionnaire and survey; 2 focus group interviews, semistructured interviews, media-related searches, and case studies to determine the level of engagement with research users, impact and processes, activities, and roles leading to impact		Not specified	None
P. Buykx et al. **The Health Services Research Impact Framework [5]** Australia, 2012		• PHC RIS [31] • RIF [29] • Exchange Model [44]	Framework for recording and monitoring the impact of health research. This framework consolidates the most relevant elements of the PHS RIS, RIF, and exchange model	• Knowledge generation and communication • Capacity building, training, and leadership • Informing policy • Improving health and health systems impact • Social and economic benefit impact	No systematic approach reported	None reported		None	Not available in the main paper
Higher Education Funding Council for England (HEFCE) **Research Assessment Exercise (RAE) [39]** UK, 2005		**–**	The aim of the RAE is to assess the quality of research conducted by academic institutions in the UK, in order to inform funding decisions by higher education funding bodies.	• RAE1: Staff information • RAE2: Research output • RAE3: Research scholarships • RAE4: Attractiveness for external funding • RAE5: Further information on groups of research	None reported	Focus groups, workshops, and meetings with HEFCE institutions, funding body officers, external experts, and stakeholder groups		Not specified	None
C. Donovan **Australian Research Quality Framework (RQF) [38]** **Substituted in 2015 by the Excellence in Research for Australia (ERA) framework** Australia, 2008		**–**	A 5-point rating scale to evaluate research excellence and societal returns of publicly funded research. This framework highlights the importance of end-users’ interactions to enhance the use of research	The impact rating scale: • Research has produced an outstanding social, economic, environmental, or cultural benefit. • Research has produced a significant social, economic, environmental, or cultural benefit. • Research has produced new policies, products, attitudes, behaviours, or outlooks in the end-user communities. • Research has engaged with the end-user community to address a social, economic, environmental, or cultural issue. • Research has had limited or no identifiable social, economic, environmental, or cultural outcome.	None reported	The technical working group on research impact (senior university managers, representatives from business and industry, experts in impact evaluation, and members of the development advisory group) and the Australian higher education sector participated during different phases of the development of the framework. The Australian higher education sector was consulted. The technical working group was in charge of further development of the framework characteristics		Not specified	None
HEFCE **Research Excellence Framework (REF 2014) [3]** UK, 2011		• RQF [38,39]	Framework for assessing the quality of research of UK higher education institutions	• REF1: Staff details • REF2: Research outputs • REF3: Impact template and case studies • REF4: Environmental data • REF5: Environmental template	Scoping study, no systematic approach reported	An initial consultation on the REF was conducted among HEFCE institutions and other stakeholders to determine the potential of introducing bibliometric indicators in the REF. A second consultation was conducted based on the results of the first initiative, which included proposals on how to assess the impact of research		Not specified	A pilot exercise was conducted to test and develop bibliometric indicators to measure research quality. A second pilot was conducted to test the proposals to assess research impact
R. Jacob and M. McGregor **Health Technology Assessment (HTA) Organisation Assessment Framework [47]** Canada, 2007		**–**	The impact of health technology assessment is measured by the ability to influence the efficiency of the healthcare system	Levels of importance according to the type of policy involved: • Level 1: General statements of ministerial policy • Level 2: Planning guidelines for health services organisation • Level 3: Practice norms prescribed by the professional corporation • Level 4: Ministry rules concerning coverage of health services • Level 5: Hospital rules concerning utilisation of services • Level 6: Ministry decisions on the organisation of specific health services • Level 7: Ministry decisions concerning the allocation of resources	No, a case study approach was undertaken to determine the impact of health technology assessment on policy decision	Interviews and questionnaires were conducted with stakeholders affiliated with the Canadian Ministry of Health; those who were involved in defining policy were contacted to corroborate the supporting documentation collected		Not specified	None
R. Landry et al. **Research Utilisation Ladder [46]** Canada, 2001		• Knott and Wildavsky Model [57]	Assessment of the pathway in which research progresses towards its utilisation by decision makers and practitioners	• Transmission • Cognition • Reference • Effort • Influence • Application	Yes, but no systematic approach reported	A mail survey and telephone calls were used to identify potential participants. Once the participants were recruited, a questionnaire focused on utilisation knowledge was distributed. The stakeholders involved were faculty members of different Canadian universities		Not specified	A modified version of the Knott and Wildavsky model was used to measure knowledge utilization from the data collected. A quantitative approach was adopted to analyse the data of the model
C. Sarli et al. **The Becker Medical Library Model for Assessment of Research Impact [36]** US, 2010		• W. K. Kellogg Foundation Model [58]	Methodology beyond citation counts, to assess research impact as a result of research output. This model allows the interaction between researchers and institutions to document and quantify the impact of research	• Research output • Knowledge transfer • Clinical implementations • Community benefit	Yes, but no systematic approach reported	Authors consulted expert opinion, researchers, clinicians, and librarians to identify indicators to measure research impact		Not specified	The evidence available to measure the impact of the Ocular Hypertension Treatment Study was analysed using the preliminary framework. The outcomes refined the framework leading to a tool to assess research impact
**V. C. Brueton et al. [37]** UK, 2014		• The Becker Model [36]	Framework used to better report and assess impact of methodological research	• Advancement of knowledge • Implementation	No systematic approach reported	Two semistructured interviews and email queries to the methodologists for the included projects were used to examine other indicators and analyse evidence of research implementation		Not specified	None
Royal Netherlands Academy of Arts and Sciences **The social impact of applied research [41]** The Netherlands, 2002		**–**	Measuring the societal impact of applied research, as an incentive for researchers to improve their performance within this field	• Science and certified knowledge • Education and training • Innovation and professionalism • Public policy • Collaboration and visibility	Yes, but not systematic approach reported	None		None	None
A. Weiss **Logic Model for Medical Research [30]** US, 2007		• United Way Model [59]	Measurement outcomes (awareness, implementation, and patient benefit) provide the basis to assess the quality of the investment in research	• Initial outcome: awareness • Intermediate outcome: implementation • Long-term outcome: patient benefit	No systematic approach reported	None		None	Qualitative methods and a case study were conducted to assess the impact of the model developed
J. Canavan et al. [45] UK, 2009		• Models of research impact: A cross sector review of literature and practice [60]	This approach proposes 6 recommendations that can increase the likelihood of an effective and practical measurement of research impact policy	Recommendations: • Commissioner-researcher partnerships • Embedded researcher model • Planning for impact from the outset • Adopting facilitative research methodological strategies • Following good practice towards research impact • Connecting research measurement to performance management	Yes, but no systematic approach reported	None		None	A case study was developed to exemplify the framework proposed
J. Spaapen and L. van Drooge **Social Impact Assessment Methods for research and funding instruments through the study of Productive Interactions between science and society (SIAMPI) [40]** The Netherlands, 2011		**–**	Learning tool to better understand how research interactions lead to social impact. This approach focuses on productive interactions, especially exchanges between researchers and stakeholders	Types of productive interactions: • Direct interactions • Indirect interactions • Financial interactions	Yes, but no systematic approach reported	The European commission, research organisations, science policy makers, research councils, academies, and other bodies involved in research were involved in meetings and discussions		A case study approach was used to test the framework. The feedback of the case study representatives was used to refine the framework [61]	Not specified
M. O. Kok and A. Schuit **Contribution Mapping [7]** The Netherlands, 2012		**–**	An approach to monitor and evaluate contributions to determine how the utilisation of research can contribute to better action for health. The method focuses on processes, actors, and efforts to enhance contributions and enable alignment of efforts.	Research related contributions categories: • Changes in abilities and actions of involved and linked actors • Contributed knowledge products • Contributions through linked utilisation • Indications of utilisation at a distance	Yes, but no systematic approach reported	Investigators of the projects were included. Potential key users and other potential informants were interviewed to draft the model and understand contributions. Stakeholders were consulted to refine the model		Preliminary results were shared with stakeholders for feedback and validation. Once discrepancies were solved, results were shared with stakeholders	Not specified
S. Morton **Research Contribution Framework [42]** UK, 2015		**–**	Assessment of research impact using contribution analysis to explain the influence in policy and practice	• Final outcome • Policy or practice change • Capacity, knowledge and skill • Awareness, reaction • Engagement, participation • Activities and outputs • Inputs	No systematic approach reported	Semistructured interviews were conducted among project partners that took part in the process of the research (i.e., conferences and workshops) and research users (i.e., practitioners)		A thematic analysis approach was used to analyse the data collected and refine the framework. The themes that emerged were tested against other sources	A case study was used to illustrate how the Research Contribution Framework can assess impact

### Methodological framework development

The included methodological frameworks varied in their development process, but there were some common approaches employed. Most included a literature review [2,5,7,8,31,33,36,37,40–46], although none of them used a recognised systematic method. Most also consulted with various stakeholders [3,8,29,31,33,35–38,43,44,46,47] but used differing methods to incorporate their views, including quantitative surveys [32,35,43,46], face-to-face interviews [7,29,33,35,37,42,43], telephone interviews [31,46], consultation [3,7,36], and focus groups [39,43]. A range of stakeholder groups were approached across the sample, including principal investigators [7,29,43], research end users [7,42,43], academics [3,8,39,40,43,46], award holders [43], experts [33,38,39], sponsors [33,39], project coordinators [32,42], and chief investigators [31,35]. However, some authors failed to identify the stakeholders involved in the development of their frameworks [2,5,34,41,45], making it difficult to assess their appropriateness. In addition, only 4 of the included papers reported using formal analytic methods to interpret stakeholder responses. These included the Canadian Academy of Health Sciences framework, which used conceptual cluster analysis [33]. The Research Contribution [42], Research Impact [29], and Primary Health Care & Information Service [31] used a thematic analysis approach. Finally, some authors went on to pilot their framework, which shaped refinements on the methodological frameworks until approval. Methods used to pilot the frameworks included a case study approach [2,3,30,32,33,36,40,42,44,45], contrasting results against available literature [29], the use of stakeholders’ feedback [7], and assessment tools [35,46].

### Major impact categories

#### 1. Primary research-related impact

A number of methodological frameworks advocated the evaluation of ‘research-related impact’. This encompassed content related to the generation of new knowledge, knowledge dissemination, capacity building, training, leadership, and the development of research networks. These outcomes were considered the direct or primary impacts of a research project, as these are often the first evidenced returns [30,62].

A number of subgroups were identified within this category, with frameworks supporting the collection of impact data across the following constructs: ‘research and innovation outcomes’; ‘dissemination and knowledge transfer’; ‘capacity building, training, and leadership’; and ‘academic collaborations, research networks, and data sharing’.

*1*.*1*. *Research and innovation outcomes*. Twenty of the 24 frameworks advocated the evaluation of ‘research and innovation outcomes’ [2,3,5,7,8,29–39,41,43,44,46]. This subgroup included the following metrics: number of publications; number of peer-reviewed articles (including journal impact factor); citation rates; requests for reprints, number of reviews, and meta-analysis; and new or changes in existing products (interventions or technology), patents, and research. Additionally, some frameworks also sought to gather information regarding ‘methods/methodological contributions’. These advocated the collection of systematic reviews and appraisals in order to identify gaps in knowledge and determine whether the knowledge generated had been assessed before being put into practice [29].

*1*.*2*. *Dissemination and knowledge transfer*. Nineteen of the 24 frameworks advocated the assessment of ‘dissemination and knowledge transfer’ [2,3,5,7,29–32,34–43,46]. This comprised collection of the following information: number of conferences, seminars, workshops, and presentations; teaching output (i.e., number of lectures given to disseminate the research findings); number of reads for published articles; article download rate and number of journal webpage visits; and citations rates in nonjournal media such as newspapers and mass and social media (i.e., Twitter and blogs). Furthermore, this impact subgroup considered the measurement of research uptake and translatability and the adoption of research findings in technological and clinical applications and by different fields. These can be measured through patents, clinical trials, and partnerships between industry and business, government and nongovernmental organisations, and university research units and researchers [29].

*1*.*3*. *Capacity building*, *training*, *and leadership*. Fourteen of 24 frameworks suggested the evaluation of ‘capacity building, training, and leadership’ [2,3,5,8,29,31–35,39–41,43]. This involved collecting information regarding the number of doctoral and postdoctoral studentships (including those generated as a result of the research findings and those appointed to conduct the research), as well as the number of researchers and research-related staff involved in the research projects. In addition, authors advocated the collection of ‘leadership’ metrics, including the number of research projects managed and coordinated and the membership of boards and funding bodies, journal editorial boards, and advisory committees [29]. Additional metrics in this category included public recognition (number of fellowships and awards for significant research achievements), academic career advancement, and subsequent grants received. Lastly, the impact metric ‘research system management’ comprised the collection of information that can lead to preserving the health of the population, such as modifying research priorities, resource allocation strategies, and linking health research to other disciplines to maximise benefits [29].

*1*.*4*. *Academic collaborations*, *research networks*, *and data sharing*. Lastly, 10 of the 24 frameworks advocated the collection of impact data regarding ‘academic collaborations (internal and external collaborations to complete a research project), research networks, and data sharing’ [2,3,5,7,29,34,37,39,41,43].

#### 2. Influence on policy making

Methodological frameworks addressing this major impact category focused on measurable improvements within a given knowledge base and on interactions between academics and policy makers, which may influence policy-making development and implementation. The returns generated in this impact category are generally considered as intermediate or midterm (1 to 3 years). These represent an important interim stage in the process towards the final expected impacts, such as quantifiable health improvements and economic benefits, without which policy change may not occur [30,62]. The following impact subgroups were identified within this category: ‘type and nature of policy impact’, ‘level of policy making’, and ‘policy networks’.

*2*.*1*. *Type and nature of policy impact*. The most common impact subgroup, mentioned in 18 of the 24 frameworks, was ‘type and nature of policy impact’ [2,7,29–38,41–43,45–47]. Methodological frameworks addressing this subgroup stressed the importance of collecting information regarding the influence of research on policy (i.e., changes in practice or terminology). For instance, a project looking at trafficked adolescents and women (2003) influenced the WHO guidelines (2003) on ethics regarding this particular group [17,21,63].

*2*.*2*. *Level of policy impact*. Thirteen of 24 frameworks addressed aspects surrounding the need to record the ‘level of policy impact’ (international, national, or local) and the organisations within a level that were influenced (local policy makers, clinical commissioning groups, and health and wellbeing trusts) [2,5,8,29,31,34,38,41,43–47]. Authors considered it important to measure the ‘level of policy impact’ to provide evidence of collaboration, coordination, and efficiency within health organisations and between researchers and health organisations [29,31].

*2*.*3*. *Policy networks*. Five methodological frameworks highlighted the need to collect information regarding collaborative research with industry and staff movement between academia and industry [5,7,29,41,43]. A policy network emphasises the relationship between policy communities, researchers, and policy makers. This relationship can influence and lead to incremental changes in policy processes [62].

#### 3. Health and health systems impact

A number of methodological frameworks advocated the measurement of impacts on health and healthcare systems across the following impact subgroups: ‘quality of care and service delivering’, ‘evidence-based practice’, ‘improved information and health information management’, ‘cost containment and effectiveness’, ‘resource allocation’, and ‘health workforce’.

*3*.*1*. *Quality of care and service delivery*. Twelve of the 24 frameworks highlighted the importance of evaluating ‘quality of care and service delivery’ [2,5,8,29–31,33–36,41,47]. There were a number of suggested metrics that could be potentially used for this purpose, including health outcomes such as quality-adjusted life years (QALYs), patient-reported outcome measures (PROMs), patient satisfaction and experience surveys, and qualitative data on waiting times and service accessibility.

*3*.*2*. *Evidence-based practice*. ‘Evidence-based practice’, mentioned in 5 of the 24 frameworks, refers to making changes in clinical diagnosis, clinical practice, treatment decisions, or decision making based on research evidence [5,8,29,31,33]. The suggested metrics to demonstrate evidence-based practice were adoption of health technologies and research outcomes to improve the healthcare systems and inform policies and guidelines [29].

*3*.*3*. *Improved information and health information management*. This impact subcategory, mentioned in 5 of the 24 frameworks, refers to the influence of research on the provision of health services and management of the health system to prevent additional costs [5,29,33,34,38]. Methodological frameworks advocated the collection of health system financial, nonfinancial (i.e., transport and sociopolitical implications), and insurance information in order to determine constraints within a health system.

*3*.*4*. *Cost containment and cost-effectiveness*. Six of the 24 frameworks advocated the subcategory ‘cost containment and cost-effectiveness’ [2,5,8,17,33,36]. ‘Cost containment’ comprised the collection of information regarding how research has influenced the provision and management of health services and its implication in healthcare resource allocation and use [29]. ‘Cost-effectiveness’ refers to information concerning economic evaluations to assess improvements in effectiveness and health outcomes—for instance, the cost-effectiveness (cost and health outcome benefits) assessment of introducing a new health technology to replace an older one [29,31,64].

*3*.*5*. *Resource allocation*. ‘Resource allocation’, mentioned in 6frameworks, can be measured through 2 impact metrics: new funding attributed to the intervention in question and equity while allocating resources, such as improved allocation of resources at an area level; better targeting, accessibility, and utilisation; and coverage of health services [2,5,29,31,45,47]. The allocation of resources and targeting can be measured through health services research reports, with the utilisation of health services measured by the probability of providing an intervention when needed, the probability of requiring it again in the future, and the probability of receiving an intervention based on previous experience [29,31].

*3*.*6*. *Health workforce*. Lastly, ‘health workforce’, present in 3 methodological frameworks, refers to the reduction in the days of work lost because of a particular illness [2,5,31].

#### 4. Health-related and societal impact

Three subgroups were included in this category: ‘health literacy’; ‘health knowledge, attitudes, and behaviours’; and ‘improved social equity, inclusion, or cohesion’.

*4*.*1*. *Health knowledge*, *attitudes*, *and behaviours*. Eight of the 24 frameworks suggested the assessment of ‘health knowledge, attitudes, behaviours, and outcomes’, which could be measured through the evaluation of levels of public engagement with science and research (e.g., National Health Service (NHS) Choices end-user visit rate) or by using focus groups to analyse changes in knowledge, attitudes, and behaviour among society [2,5,29,33–35,38,43].

*4*.*2*. *Improved equity*, *inclusion*, *or cohesion and human rights*. Other methodological frameworks, 4 of the 24, suggested capturing improvements in equity, inclusion, or cohesion and human rights. Authors suggested these could be using a resource like the United Nations Millennium Development Goals (MDGs) (superseded by Sustainable Development Goals [SDGs] in 2015) and human rights [29,33,34,38]. For instance, a cluster-randomised controlled trial in Nepal, which had female participants, has demonstrated the reduction of neonatal mortality through the introduction of maternity health care, distribution of delivery kits, and home visits. This illustrates how research can target vulnerable and disadvantaged groups. Additionally, this research has been introduced by the World Health Organisation to achieve the MDG ‘improve maternal health’ [16,29,65].

*4*.*3*. *Health literacy*. Some methodological frameworks, 3 of the 24, focused on tracking changes in the ability of patients to make informed healthcare decisions, reduce health risks, and improve quality of life, which were demonstrably linked to a particular programme of research [5,29,43]. For example, a systematic review showed that when HIV health literacy/knowledge is spread among people living with the condition, antiretroviral adherence and quality of life improve [66].

#### 5. Broader economic impacts

Some methodological frameworks, 9 of 24, included aspects related to the broader economic impacts of health research—for example, the economic benefits emerging from the commercialisation of research outputs [2,5,29,31,33,35,36,38,67]. Suggested metrics included the amount of funding for research and development (R&D) that was competitively awarded by the NHS, medical charities, and overseas companies. Additional metrics were income from intellectual property, spillover effects (any secondary benefit gained as a repercussion of investing directly in a primary activity, i.e., the social and economic returns of investing on R&D) [33], patents granted, licences awarded and brought to the market, the development and sales of spinout companies, research contracts, and income from industry.

The benefits contained within the categories ‘health and health systems impact’, ‘health-related and societal impact’, and ‘broader economic impacts’ are considered the expected and final returns of the resources allocated in healthcare research [30,62]. These benefits commonly arise in the long term, beyond 5 years according to some authors, but there was a recognition that this could differ depending on the project and its associated research area [4].

### Data synthesis

**Fig 2:**
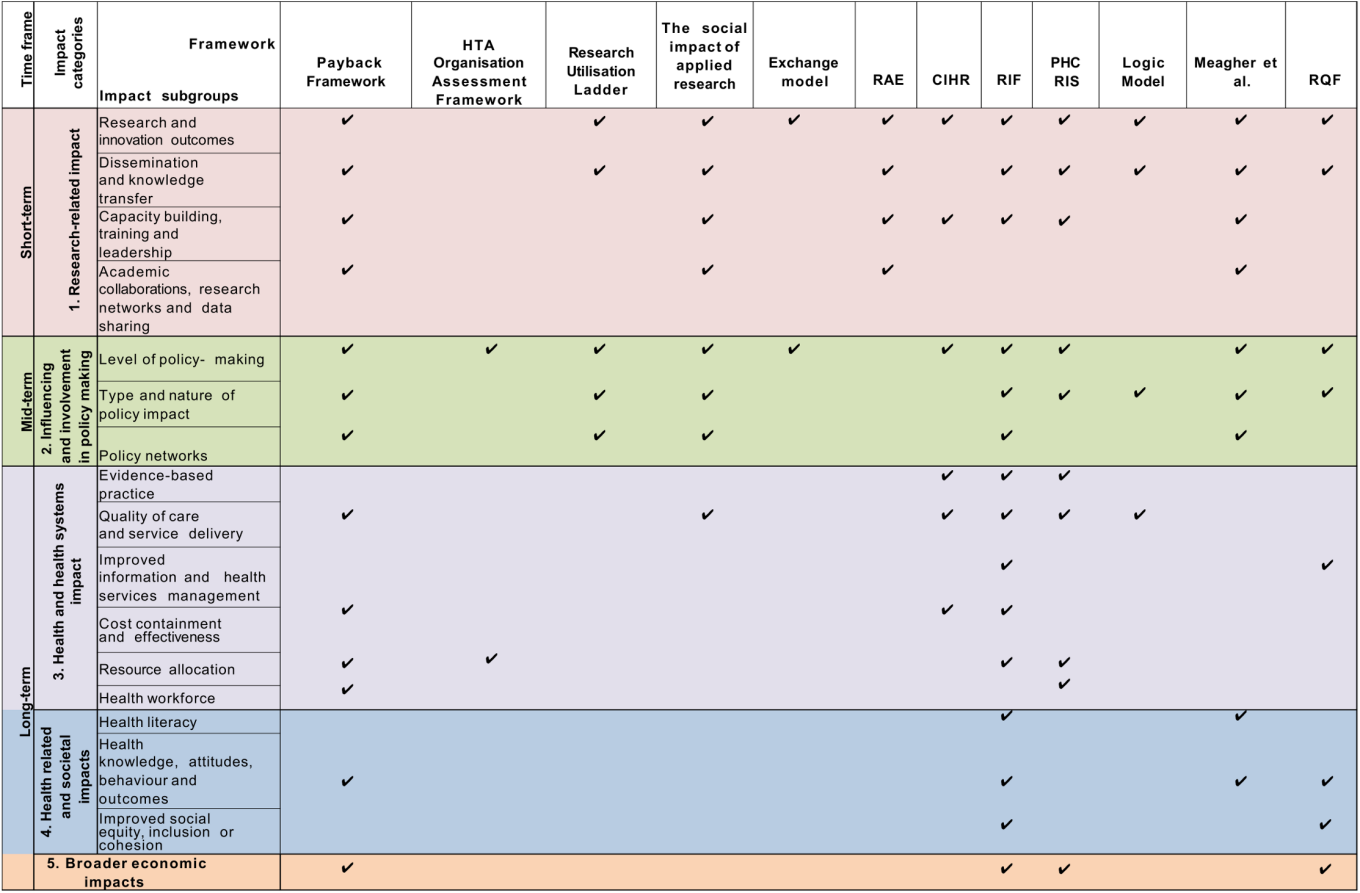
The impact matrix (1). CIHR, Canadian Institutes of Health Research; HTA, Health Technology Assessment; PHC RIS, Primary Health Care Research & Information Service; RAE, Research Assessment Exercise; RQF, Research Quality Framework.

**Fig 3:**
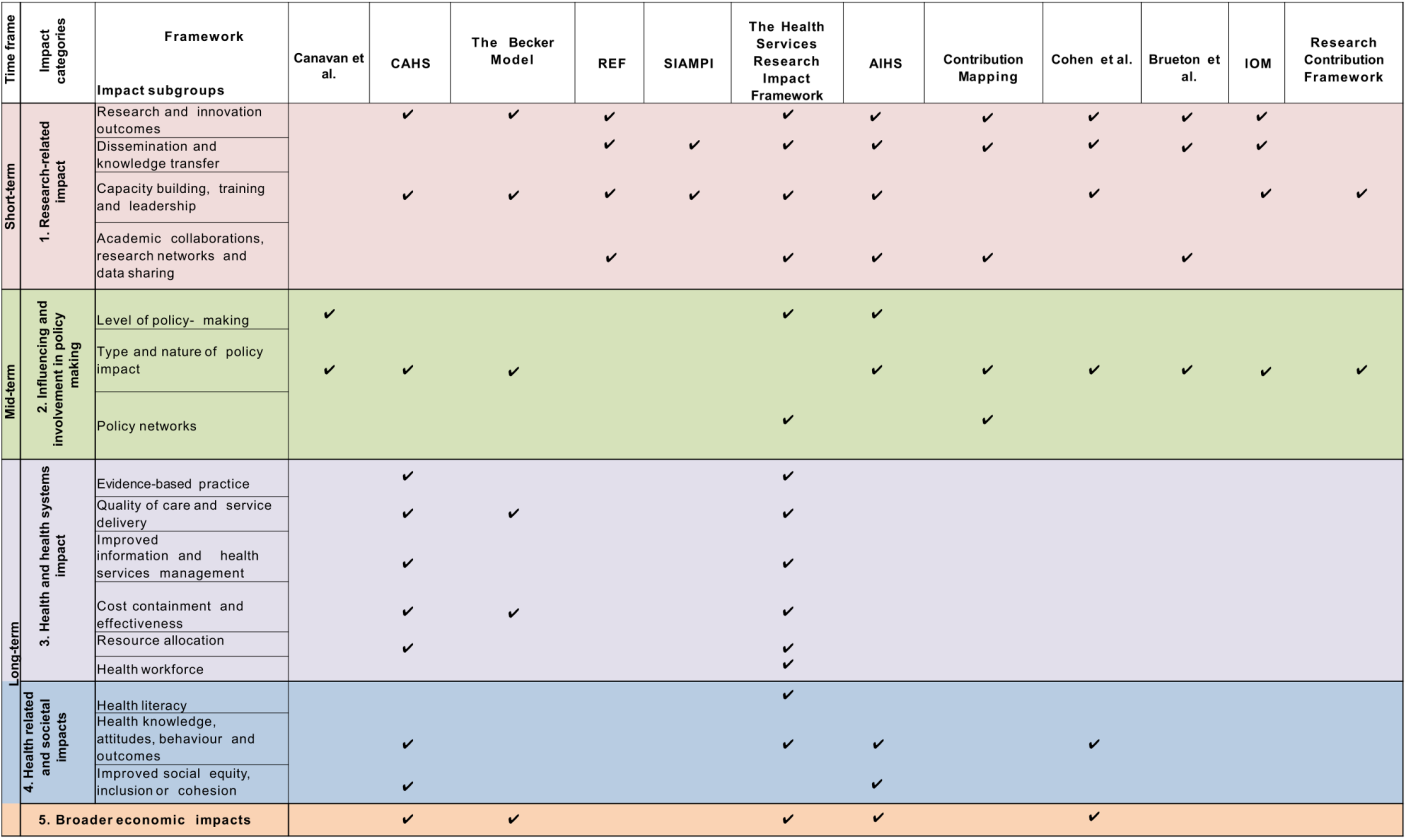
The impact matrix (2). AIHS, Alberta Innovates—Health Solutions; CAHS, Canadian Institutes of Health Research; IOM, Impact Oriented Monitoring; REF, Research Excellence Framework; SIAMPI, Social Impact Assessment Methods for research and funding instruments through the study of Productive Interactions between science and society.

Five major impact categories were identified across the 24 included methodological frameworks: (1) ‘primary research-related impact’, (2) ‘influence on policy making’, (3) ‘health and health systems impact’, (4) ‘health-related and societal impact’, and (5) ‘broader economic impact’. These major impact categories were further subdivided into 16 impact subgroups. The included publications proposed 80 different metrics to measure research impact. This impact typology synthesis is depicted in ‘the impact matrix’ (Fig 2 and Fig 3).

### Commonality and differences across frameworks

The ‘Research Impact Framework’ and the ‘Health Services Research Impact Framework’ were the models that encompassed the largest number of the metrics extracted. The most dominant methodological framework was the Payback Framework; 7 other methodological framework models used the Payback Framework as a starting point for development [8,29,31–35]. Additional methodological frameworks that were commonly incorporated into other tools included the CIHR framework, the CAHS model, the AIHS framework, and the Exchange model [8,33,34,44]. The capture of ‘research-related impact’ was the most widely advocated concept across methodological frameworks, illustrating the importance with which primary short-term impact outcomes were viewed by the included papers. Thus, measurement of impact via number of publications, citations, and peer-reviewed articles was the most common. ‘Influence on policy making’ was the predominant midterm impact category, specifically the subgroup ‘type and nature of policy impact’, in which frameworks advocated the measurement of (i) changes to legislation, regulations, and government policy; (ii) influence and involvement in decision-making processes; and (iii) changes to clinical or healthcare training, practice, or guidelines. Within more long-term impact measurement, the evaluations of changes in the ‘quality of care and service delivery’ were commonly advocated.

**Fig 4:**
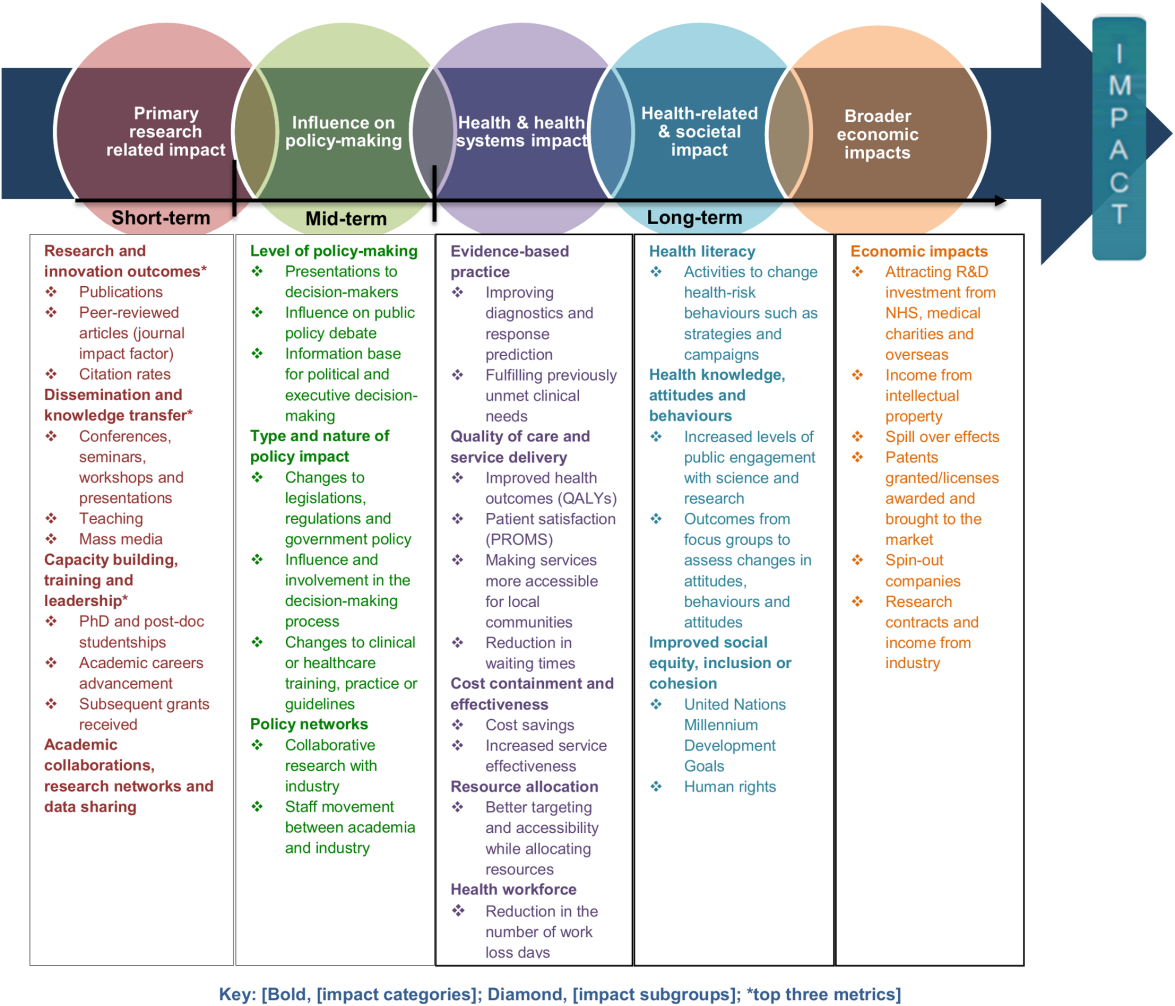
Pathways to research impact. NHS, National Health Service; PROM, patient-reported outcome measure; QALY, quality-adjusted life year; R&D, research and development.

In light of the commonalities and differences among the methodological frameworks, the ‘pathways to research impact’ diagram (Fig 4) was developed to provide researchers, funders, and policy makers a more comprehensive and exhaustive way to measure healthcare research impact. The diagram has the advantage of assorting all the impact metrics proposed by previous frameworks and grouping them into different impact subgroups and categories. Prospectively, this global picture will help researchers, funders, and policy makers plan strategies to achieve multiple pathways to impact before carrying the research out. The analysis of the data extraction and construction of the impact matrix led to the development of the ‘pathways to research impact’ diagram (Fig 4). The diagram aims to provide an exhaustive and comprehensive way of tracing research impact by combining all the impact metrics presented by the different 24 frameworks, grouping those metrics into different impact subgroups, and grouping these into broader impact categories.

## Discussion

This review has summarised existing methodological impact frameworks together for the first time using systematic methods (Fig 4). It allows researchers and funders to consider pathways to impact at the design stage of a study and to understand the elements and metrics that need to be considered to facilitate prospective assessment of impact. Users do not necessarily need to cover all the aspects of the methodological framework, as every research project can impact on different categories and subgroups. This review provides information that can assist researchers to better demonstrate impact, potentially increasing the likelihood of conducting impactful research and reducing research waste. Existing reviews have not presented a methodological framework that includes different pathways to impact, health impact categories, subgroups, and metrics in a single methodological framework.

Academic-orientated frameworks included in this review advocated the measurement of impact predominantly using so-called ‘quantitative’ metrics—for example, the number of peer-reviewed articles, journal impact factor, and citation rates. This may be because they are well-established measures, relatively easy to capture and objective, and are supported by research funding systems. However, these metrics primarily measure the dissemination of research finding rather than its impact [30,68]. Whilst it is true that wider dissemination, especially when delivered via world-leading international journals, may well lead eventually to changes in healthcare, this is by no means certain. For instance, case studies evaluated by Flinders University of Australia demonstrated that some research projects with non-peer-reviewed publications led to significant changes in health policy, whilst the studies with peer-reviewed publications did not result in any type of impact [68]. As a result, contemporary literature has tended to advocate the collection of information regarding a variety of different potential forms of impact alongside publication/citations metrics [2,3,5,7,8,29–47], as outlined in this review.

The 2014 REF exercise adjusted UK university research funding allocation based on evidence of the wider impact of research (through case narrative studies and quantitative metrics), rather than simply according to the quality of research [12]. The intention was to ensure funds were directed to high-quality research that could demonstrate actual realised benefit. The inclusion of a mixed-method approach to the measurement of impact in the REF (narrative and quantitative metrics) reflects a widespread belief—expressed by the majority of authors of the included methodological frameworks in the review—that individual quantitative impact metrics (e.g., number of citations and publications) do not necessary capture the complexity of the relationships involved in a research project and may exclude measurement of specific aspects of the research pathway [10,12].

Many of the frameworks included in this review advocated the collection of a range of academic, societal, economic, and cultural impact metrics; this is consistent with recent recommendations from the Stern review [10]. However, a number of these metrics encounter research ‘lag’: i.e., the time between the point at which the research is conducted and when the actual benefits arise [69]. For instance, some cardiovascular research has taken up to 25 years to generate impact [70]. Likewise, the impact may not arise exclusively from a single piece of research. Different processes (such as networking interactions and knowledge and research translation) and multiple individuals and organisations are often involved [4,71]. Therefore, attributing the contribution made by each of the different actors involved in the process can be a challenge [4]. An additional problem associated to attribution is the lack of evidence to link research and impact. The outcomes of research may emerge slowly and be absorbed gradually. Consequently, it is difficult to determine the influence of research in the development of a new policy, practice, or guidelines [4,23].

A further problem is that impact evaluation is conducted ‘ex post’, after the research has concluded. Collecting information retrospectively can be an issue, as the data required might not be available. ‘ex ante’ assessment is vital for funding allocation, as it is necessary to determine the potential forthcoming impact before research is carried out [69]. Additionally, ex ante evaluation of potential benefit can overcome the issues regarding identifying and capturing evidence, which can be used in the future [4]. In order to conduct ex ante evaluation of potential benefit, some authors suggest the early involvement of policy makers in a research project coupled with a well-designed strategy of dissemination [40,69].

Providing an alternate view, the authors of methodological frameworks such as the SIAMPI, Contribution Mapping, Research Contribution, and the Exchange model suggest that the problems of attribution are a consequence of assigning the impact of research to a particular impact metric [7,40,42,44]. To address these issues, these authors propose focusing on the contribution of research through assessing the processes and interactions between stakeholders and researchers, which arguably take into consideration all the processes and actors involved in a research project [7,40,42,43]. Additionally, contributions highlight the importance of the interactions between stakeholders and researchers from an early stage in the research process, leading to a successful ex ante and ex post evaluation by setting expected impacts and determining how the research outcomes have been utilised, respectively [7,40,42,43]. However, contribution metrics are generally harder to measure in comparison to academic-orientated indicators [72].

Currently, there is a debate surrounding the optimal methodological impact framework, and no tool has proven superior to another. The most appropriate methodological framework for a given study will likely depend on stakeholder needs, as each employs different methodologies to assess research impact [4,37,41]. This review allows researchers to select individual existing methodological framework components to create a bespoke tool with which to facilitate optimal study design and maximise the potential for impact depending on the characteristic of their study (Fig 2 and Fig 3). For instance, if researchers are interested in assessing how influential their research is on policy making, perhaps considering a suite of the appropriate metrics drawn from multiple methodological frameworks may provide a more comprehensive method than adopting a single methodological framework. In addition, research teams may wish to use a multidimensional approach to methodological framework development, adopting existing narratives and quantitative metrics, as well as elements from contribution frameworks. This approach would arguably present a more comprehensive method of impact assessment; however, further research is warranted to determine its effectiveness [4,69,72,73].

Finally, it became clear during this review that the included methodological frameworks had been constructed using varied methodological processes. At present, there are no guidelines or consensus around the optimal pathway that should be followed to develop a robust methodological framework. The authors believe this is an area that should be addressed by the research community, to ensure future frameworks are developed using best-practice methodology.

For instance, the Payback Framework drew upon a literature review and was refined through a case study approach. Arguably, this approach could be considered inferior to other methods that involved extensive stakeholder involvement, such as the CIHR framework [8]. Nonetheless, 7 methodological frameworks were developed based upon the Payback Framework [8,29,31–35].

### Limitations

The present review is the first to summarise systematically existing impact methodological frameworks and metrics. The main limitation is that 50% of the included publications were found through methods other than bibliographic databases searching, indicating poor indexing. Therefore, some relevant articles may not have been included in this review if they failed to indicate the inclusion of a methodological impact framework in their title/abstract. We did, however, make every effort to try to find these potentially hard-to-reach publications, e.g., through forwards/backwards citation searching, hand searching reference lists, and expert communication. Additionally, this review only extracted information regarding the methodology followed to develop each framework from the main publication source or framework webpage. Therefore, further evaluations may not have been included, as they are beyond the scope of the current paper. A further limitation was that although our search strategy did not include language restrictions, we did not specifically search non-English language databases. Thus, we may have failed to identify potentially relevant methodological frameworks that were developed in a non-English language setting.

### Conclusion

In conclusion, the measurement of research impact is an essential exercise to help direct the allocation of limited research resources, to maximise benefit, and to help minimise research waste. This review provides a collective summary of existing methodological impact frameworks and metrics, which funders may use to inform the measurement of research impact and researchers may use to inform study design decisions aimed at maximising the short-, medium-, and long-term impact of their research.

## Supporting information

S1 Appendix Search strategy. (TIF)

S1 PRISMA Checklist Preferred Reporting Items for Systematic Reviews and Meta-Analyses (PRISMA) checklist. (PDF)

## Abbreviations

AIHS: Alberta Innovates—Health Solutions
CAHS: Canadian Academy of Health Sciences
CIHR: Canadian Institutes of Health Research
CINAHL+: Cumulative Index to Nursing and Allied Health Literature
EMBASE: Excerpta Medica Database
ERA: Excellence in Research for Australia
HEFCE: Higher Education Funding Council for England
HMIC: Health Management Information Consortium
HTA: Health Technology Assessment
IOM: Impact Oriented Monitoring
MDG: Millennium Development Goal
NHS: National Health Service
MEDLINE: Medical Literature Analysis and Retrieval System Online
PHC RIS: Primary Health Care Research & Information Service
PRISMA: Preferred Reporting Items for Systematic Reviews and Meta-Analyses
PROM: patient-reported outcome measures
QALY: quality-adjusted life year
R&D: research and development
RAE: Research Assessment Exercise
REF: Research Excellence Framework
RIF: Research Impact Framework
RQF: Research Quality Framework
SDG: Sustainable Development Goal
SIAMPI: Social Impact Assessment Methods for research and funding instruments through the study of Productive Interactions between science and society
