# Supplementary figures and images for: Assessing the impact of healthcare research: A systematic review of methodological frameworks
Source: PLoS Med. 2017 Aug 9;14(8):e1002370. doi: 10.1371/journal.pmed.1002370 (PMC5549933; doi:10.1371/journal.pmed.1002370)

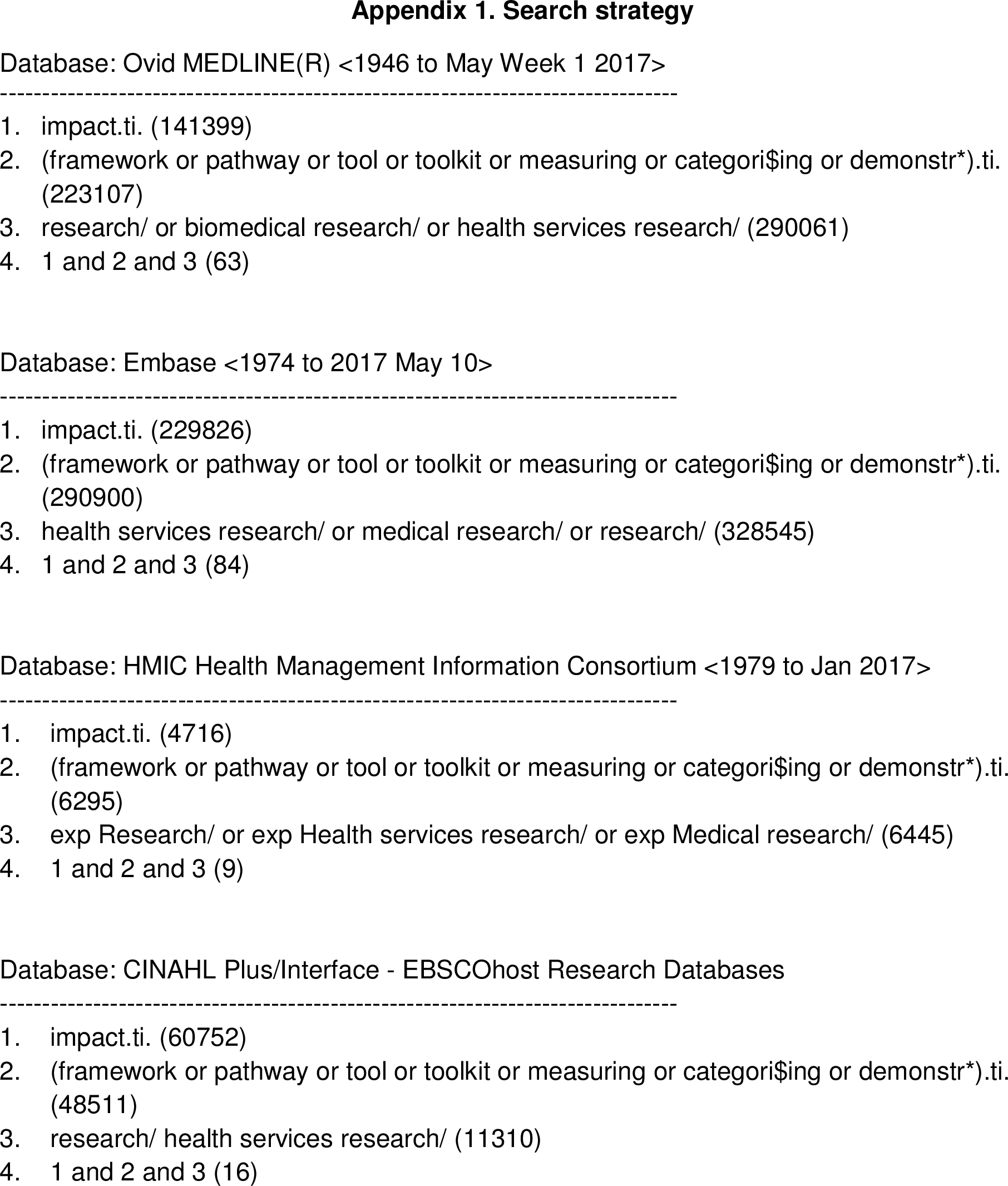

Supplement: S1 Appendix — (TIF) [file pmed.1002370.s001.tif]
